# Author Reply to: Empowering Without Misinforming Adolescents and Young Adults with Cystic Fibrosis. Comment on “Perceptions of Social Media Use to Augment Health Care Among Adolescents and Young Adults With Cystic Fibrosis: Survey Study”

**DOI:** 10.2196/39450

**Published:** 2022-05-25

**Authors:** Ryan C Perkins, Gregory S Sawicki

**Affiliations:** 1 Division of Pulmonary Medicine Boston Children's Hospital Boston, MA United States

**Keywords:** Cystic fibrosis, Social media, mobile health, adherence, adolescents, young adults, Medical misinformation

We would like to thank the authors for their thoughtful comments on our study [1]. The COVID-19 pandemic has forced us to consider the incorporation of alternative models of care delivery. As noted in our study [2], social media is a model with potential to address compliance, social support, reduce isolation, and other vulnerabilities. The authors raise the question about medical misinformation and the potential adverse implications it may impart in care delivery. The COVID-19 pandemic has illuminated these potential perils, as evidenced by the dissemination of invalid therapeutic and preventative medications to combat the SARS-CoV-2 virus [3].

With regard to cystic fibrosis, the authors highlight that 55% of respondents rarely or never checked the accuracy of medical information, suggesting a potential vulnerability of the population to medical misinformation. Although not directly assessed in our study, an underlying theme of concern regarding medical misinformation does exist. For example, 92% of respondents suggested it was important that medical information comes from well-known sources. Additionally, 92% strongly agreed or agreed that medical information should come from a trusted source like the CF Foundation and 90% strongly agreed or agreed that it should come from a physician. These findings suggest respondents did have some degree of concern surrounding misinformation although additional exploration is warranted.

The authors suggest that patients with cystic fibrosis may be more vulnerable to misinformation depending on their age. Considering the implications of age on vulnerability to misinformation is also very important. Although this population may be younger than other chronic disease cohorts [4-6], patients with CF are aging (mean age in the United States: 23.3 years) and over half of the population (57% in the United States and the United Kingdom) are older than 18 years [7,8]. It should be considered that many of these patients grew up during the rise of social media popularity. These experiences may allow for improved digital literacy and for misinformation to be more readily identified. Fake news and misinformation target consumers from all age groups; however, older adults have been noted to share more misinformation than younger users [9]. The implications of age and prior digital media experience with respect to misinformation warrants further investigation.

We agree that the voice of health care professionals will play an important role in ensuring that our patients receive accurate medical information. Although some suggestions for combating medical information have been previously proposed [10,11], it will be important as a medical community to develop frameworks for addressing misinformation. Further investigations are needed to better characterize such activities in the future.

## Abbreviations

CF: cystic fibrosis

## References

[1] Thumber NK, Bhandari P (2022). Empowering without misinforming adolescents and young adults with cystic fibrosis. Comment on “Perceptions of social media use to augment health care among adolescents and young adults with cystic fibrosis: survey study”. JMIR Pediatrics and Parenting.

[2] Perkins RC, Gross R, Regan K, Bishay L, Sawicki GS (2021). Perceptions of social media use to augment health care among adolescents and young adults with cystic fibrosis: survey study. JMIR Pediatr Parent.

[3] Ferrara E, Cresci S, Luceri L (2020). Misinformation, manipulation, and abuse on social media in the era of COVID-19. J Comput Soc Sci.

[4] Groenewegen A, Rutten FH, Mosterd A, Hoes AW (2020). Epidemiology of heart failure. Eur J Heart Fail.

[5] Khan MAB, Hashim MJ, King JK, Govender RD, Mustafa H, Al Kaabi Juma (2020). Epidemiology of type 2 diabetes - global burden of disease and forecasted trends. J Epidemiol Glob Health.

[6] Hill NR, Fatoba ST, Oke JL, Hirst JA, O'Callaghan CA, Lasserson DS, Hobbs FDR (2016). Global prevalence of chronic kidney disease - a systematic review and meta-analysis. PLoS One.

[7] Cystic Fibrosis Foundation Patient Registry 2020 Annual Data Report.

[8] UK Cystic Fibrosis Registry Annual Data Report 2020: at a glance.

[9] Brashier NM, Schacter DL (2020). Aging in an era of fake news. Curr Dir Psychol Sci.

[10] Trethewey SP (2020). Strategies to combat medical misinformation on social media. Postgrad Med J.

[11] Bautista JR, Zhang Y, Gwizdka J (2021). Healthcare professionals' acts of correcting health misinformation on social media. Int J Med Inform.

